# FuzPred: a web server for the sequence-based prediction of the context-dependent binding modes of proteins

**DOI:** 10.1093/nar/gkad214

**Published:** 2023-03-29

**Authors:** Andras Hatos, João M C Teixeira, Susana Barrera-Vilarmau, Attila Horvath, Silvio C E Tosatto, Michele Vendruscolo, Monika Fuxreiter

**Affiliations:** Department of Biomedical Sciences, University of Padova, Padova, Italy; Department of Oncology, Lausanne University Hospital, Lausanne 1011, Switzerland; Department of Computational Biology, University of Lausanne, Lausanne 1015, Switzerland; Swiss Institute of Bioinformatics, Lausanne1015, Switzerland; Swiss Cancer Center Leman, Lausanne 1011, Switzerland; Department of Biomedical Sciences, University of Padova, Padova, Italy; Department of Biomedical Sciences, University of Padova, Padova, Italy; John Curtin School of Medical Research, The Australian National University, Acton, Australia; Department of Biomedical Sciences, University of Padova, Padova, Italy; Centre for Misfolding Diseases, Department of Chemistry, University of Cambridge, UK; Department of Biomedical Sciences, University of Padova, Padova, Italy; Department of Physics and Astronomy, University of Padova, Padova, Italy

## Abstract

Proteins form complex interactions in the cellular environment to carry out their functions. They exhibit a wide range of binding modes depending on the cellular conditions, which result in a variety of ordered or disordered assemblies. To help rationalise the binding behavior of proteins, the FuzPred server predicts their sequence-based binding modes without specifying their binding partners. The binding mode defines whether the bound state is formed through a disorder-to-order transition resulting in a well-defined conformation, or through a disorder-to-disorder transition where the binding partners remain conformationally heterogeneous. To account for the context-dependent nature of the binding modes, the FuzPred method also estimates the multiplicity of binding modes, the likelihood of sampling multiple binding modes. Protein regions with a high multiplicity of binding modes may serve as regulatory sites or hot-spots for structural transitions in the assembly. To facilitate the interpretation of the predictions, protein regions with different interaction behaviors can be visualised on protein structures generated by AlphaFold. The FuzPred web server (https://fuzpred.bio.unipd.it) thus offers insights into the structural and dynamical changes of proteins upon interactions and contributes to development of structure-function relationships under a variety of cellular conditions.

## Graphical Abstract

**Graphical Abstract:**
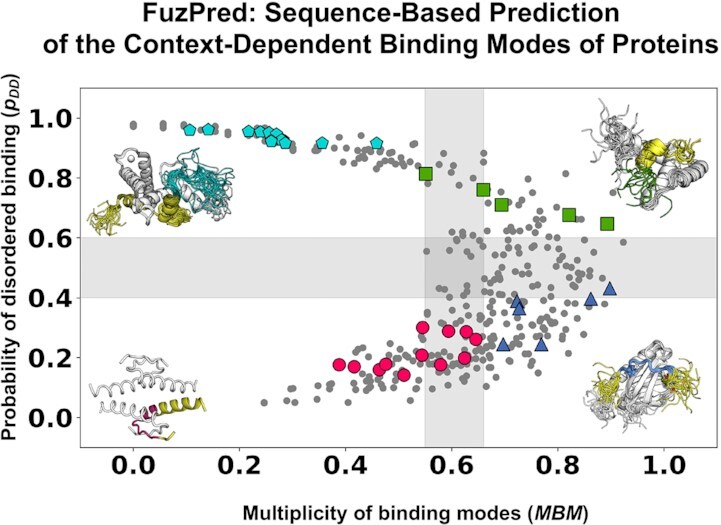
FuzPred: a web server for the sequence-based prediction of the context-dependent binding modes of proteins.

## INTRODUCTION

**Figure 1.**
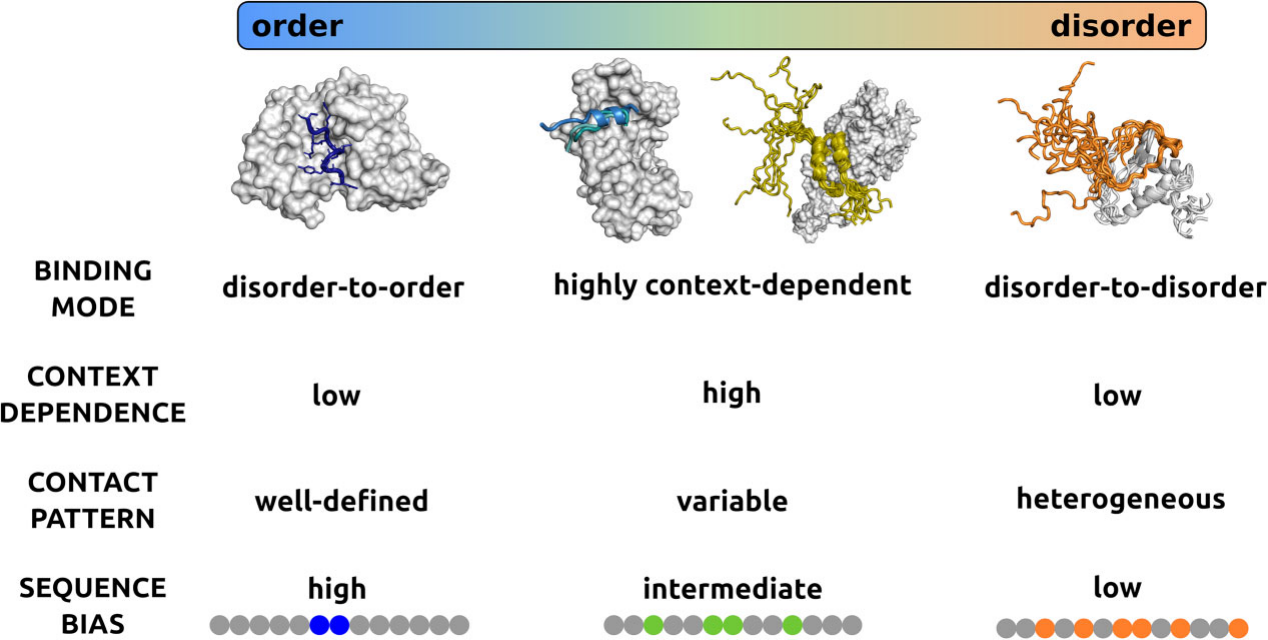
Binding modes of protein interactions. Protein interactions sample a wide range of binding modes from ordered to disordered binding. The different binding modes are characterised by structural order or disorder in the bound state (horizontal bar), which are reflected by the heterogeneity of contact patterns. In ordered binding the contact patterns are well-defined, while in disordered binding they are heterogeneous, and in case of context-dependent binding, they can be either well-defined or variable depending on the cellular conditions. At the ends of the binding mode spectrum, proteins preferentially sample a unique binding mode, therefore the context dependence is weak. In the central region of the spectrum, protein regions can sample both ordered and disordered binding modes, so the context-dependence is high. Ordered binding is generated by a strong local sequence bias (blue), whereas disordered binding is due a weak sequence bias (orange). In context-dependent binding modes, the sequence bias is modulated by the conditions, and can be strong or weak depending on the context (green). Ordered binding modes, represented by the complex of merozoite surface protein 2 (blue, top left) from *Plasmodium falciparum* and the monoclonal antibody m6D8 (gray, PDB:4qxt (41)). Disordered binding is represented by the interactions between leukemia fusion protein AF9 (gray) and elongation factor AF4 (orange) (PDB:2lm0 (42), top right), where both partners retain considerable conformational heterogeneity in the bound complex. Context-dependent binding can be achieved by polymorphism, i.e. adopting different secondary structures upon binding such as in the case of ribosomal S6 kinase 1 (marine, slate, second to left) binding to S100b (gray, PDB:5csf, 5csi, 5csj (43)) or retaining conformational disorder in a condition-dependent manner such as in the case of p150 subunit of the eukaryotic initiation factor 4F (yellow) binding to the translation initiation factor 4E, (gray PDB: 1rf8 (44)).

After the initial discovery of complexes formed by intrinsically disordered proteins, there is a recognition that proteins can sample a wide range of states in their bound forms, ranging from ordered to disordered assemblies (Figure 1) (1). Although disordered complexes were initially considered as non-specific or non-functional, ample experimental evidence based on biophysics, structure analysis, functional mutagenesis demonstrates that protein regions that remain conformationally heterogeneous in their specific complexes contribute to a wide-range of biological activities (2,3). Advances in structure determination techniques, in particular solution and single molecule methods, are enabling the characterisation of the conformational heterogeneity of disordered complexes (Figure 1) (4).

It is also becoming evident that different binding modes are associated with distinct biological functions. Disordered regions that undergo disorder-to-order transitions and adopt well-defined structures upon binding usually serve as recognition elements, which can be identified based on transient conformations in their unbound forms. For example, the tumor suppressor p53 binds to Mdm2 ubiquitin ligase through a short α-helical segment, which can also be observed in solution (Figure 1) (5,6). Protein regions that remain to be heterogeneous in the bound state usually coordinate different activities or pathway components as well as organise higher-order assemblies (7). The formation and regulation of different kinds of higher-order protein structures, from amyloid fibrils to signaling assemblies and liquid-like condensates, are all associated with conformationally heterogeneous or fuzzy regions (7). For example, in the assembly of the AIM2 inflammasome, the linker region between the PYD and CARD domains serves as a switch to expose these domains for intermolecular interactions (8). The disorder-to-disorder binding mode provides a key contribution to protein phase separation (9,10).

The complexity of interactions is underscored by sampling multiplicity of binding modes under different cellular conditions. In particular, protein regions can be induced to adopt an ordered structure upon binding, while can also remain heterogeneous under different conditions (Figure 1). Actin polymerisation, for example, is assisted by WH2 domains, which remains partly disordered upon interactions (11). WH2 domains are anchored by a single salt bridge, the stability of which is modulated by ionic strength. High ion concentrations weaken the charge interactions and increase mobility, leading to the departure of WH2 domains and elongation of the actin chain (12). At low ionic strength, in contrast, the salt bridge stabilises the complex with the actin monomer leading to sequestration. Structural examples representing different binding modes can be found in the Protein Data Bank (PDB) (13).

In this article, we describe the FuzPred web server (https://fuzpred.bio.unipd.it), which provides two key sequence-based predictions concerning the interaction behavior of proteins (Figure 1): (i) the probability to undergo disorder-to-order transition or disorder-to-disorder transitions and (ii) the likelihood of sampling a multiplicity of binding modes. The web server thus provides insights into the spectrum of interactions underlying the complex cellular behaviors of proteins.

## PREDICTION OF THE BINDING MODES OF PROTEINS

### Local sequence complexity determines the binding mode

Analysis of over 2000 specific protein complexes showed that the degree of order upon binding is weakly correlated to the presence of secondary structure elements (13). Importantly, different binding modes are associated with distinct contact patterns. The disorder-to-order binding mode is associated with well-defined contacts, formed by residues with physico-chemical features distinct from their flanking residues (13). In contrast, disorder-to-disorder binding modes are characterised by heterogeneous, alternative contact patterns (14), formed by a set of residues that can establish chemically similar interactions. For example, highly polar and charged transcription factors can bind in shallow, hydrophobic clefts of their transactivators via multiple configurations, anchored by a few hydrophobic residues (15). The KIX domain of CREB-binding protein (CBP), for example, interacts with the kinase inducible domain (KID) of the cAMP response element binding protein CREB as well as in the interactions of cMyb transcription factor in disorder-to-disorder binding mode (16). In general, the binding modes reflect the entropy change upon binding, in particular disorder-to-order binding mode corresponds to decreasing entropy upon interactions. Therefore, the binding modes are applicable to both structured and disordered protein regions.

**Figure 2.**
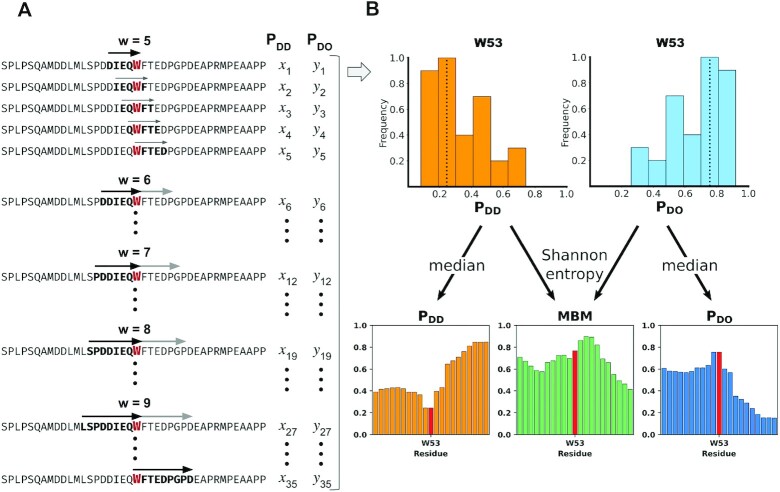
Schematic representation of the multiplicity of binding modes (MBM) calculation. (**A**) Representation of the putative binding sites. The p53 (UniProt code: P04637) transactivation (TAD) region can interact through various binding sites, the size and precise location of which are not known a priori. To predict the binding features of residue W53, for example, we consider different possible binding sites with size varying between five and nine residues. The local sequence bias is determined for all possible binding sites (windows), at all possible locations involving W53 (left panel). (**B**) Binding mode distribution analysis. This procedure generates the distribution of disorder-to-order (blue, right top) and disorder-to-disorder (orange, right top) binding modes as computed from 35 p_DO_ and p_DD_ values. The median of such p_DO_ and p_DD_ distributions provides the most likely p_DO_ and p_DD_ value, based on which the binding mode can be assigned (13). According to these predictions, W53 is likely to undergo disorder-to-order transition upon binding. The information content of the p_DO_ and p_DD_ distributions, which can be computed as a Shannon entropy, informs on the likelihood to change the binding mode with the conditions (18). This is represented by the multiplicity of binding mode (MBM) graph shown on the right bottom, middle panel), where the Shannon entropy is normalised into [0,1] range (14). The MBM graph indicates that W53 likely changes binding modes and can possibly serve as an aggregation hot-spot.

We evaluate the local sequence composition of the interaction site, which determines the binding mode (13), based on the difference between the composition of the putative interacting motif and its flanking sequence, defined as a local sequence bias (Figure 1) (13). A few residues can generate a strong ordering bias in the binding site, leading to a well-defined structure and contact pattern. In contrast, similar residues or repetitive motifs can generate a weak bias leading to heterogeneous bound states, realised via a multiplicity of bound configurations and ambiguous contact patterns. These binding modes can be predicted based on the local sequence bias, which can be evaluated without considering a specific partner (13). The sequence bias is evaluated using a window of five to nine residues representing a putative binding region (Figure 2A). To this end, the differences in the frequencies of the 20 amino acids in the binding window are combined and compared to the 20-residue N- and C-terminal flanking sequences (13). Similarly, the sum of the differences in the Kyte–Doolittle hydrophobicity are computed for the same flanking regions. In addition, the difference in tendencies to form a well-defined or disordered structure are also evaluated. The method was trained using a logistic regression model with a scoring function comprising three terms derived from the differences between the binding window and its flanking sequence, as previously described in detail (13). The performance to discriminate between disorder-to-order and disorder-to-disorder binding modes was evaluated over 2000 protein complexes resulting in an area under the curve (AUC) of 0.85 using all PDB data, and 0.92 using protein regions, which are represented at least in three complexes (13).

### Evaluating the multiplicity of binding modes (MBM)

Small variations in the location of the binding site can lead to considerable changes in the local bias (14,17). These variations can be caused by post-translational modifications, availability of further binding partners, or the presence of co-factors, metabolites or ions. The impact of such variations on the binding mode can be evaluated using multiple potential binding sites with different length and position around the same residue (Figure 2A). This provides a distribution of binding modes, which are available for interactions of a given residue (Figure 2B) (18). The median of such distribution characterises the most likely binding mode for a given residue (18). The width of the distribution informs on the likelihood of sampling multiple binding modes. The MBM is quantified by the Shannon entropy computed from the binding mode distribution (Figure 2B) (18). The MBM characterises context-dependence, the predicted impact of the cellular environment on the binding mode (19,20). The FuzPred method performs AUC of over 0.90 in distinguishing context-dependent regions (CDR) from disorder-to-order and disorder-to-disorder regions using 750 protein complexes (18).

### The interaction behavior is represented by a binding mode landscape

**Figure 3.**
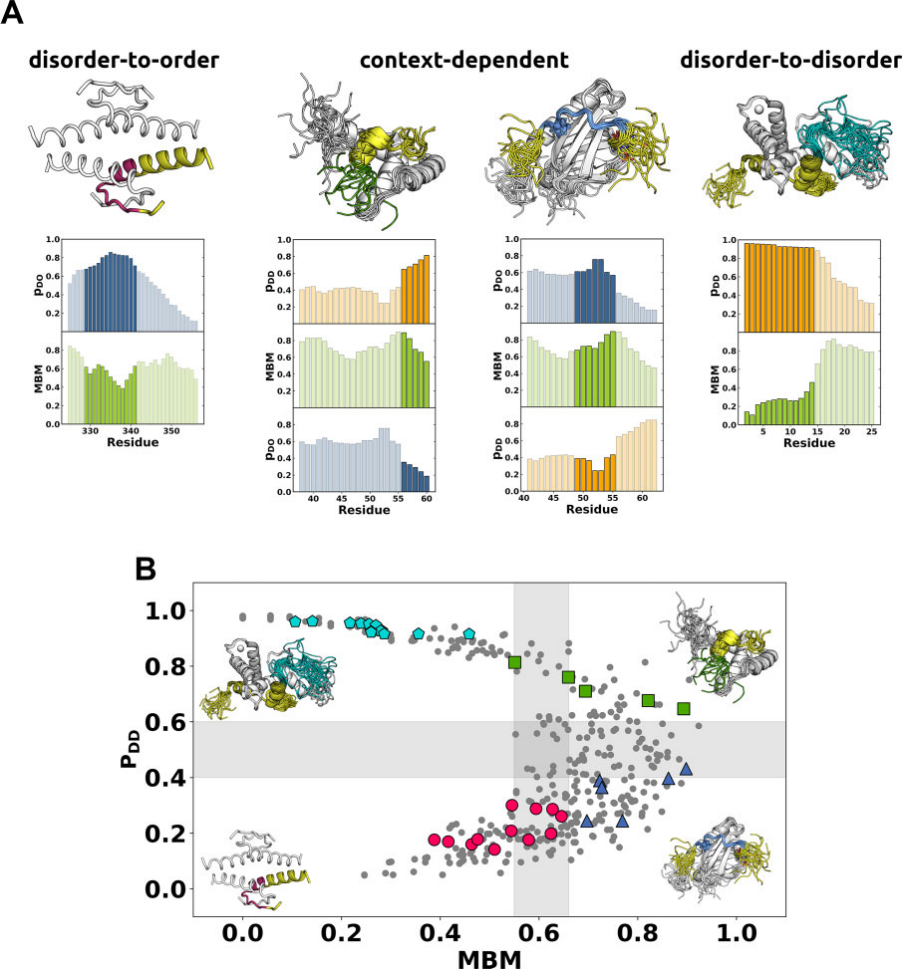
FuzPred predictions of different interaction behaviors of p53. (**A**) Binding mode prediction. The binding modes are classified based on the probabilities of disorder-to-order (p_DO_, blue, graphs) and disorder-to-disorder transitions (p_DD_, orange graphs); and the multiplicity of binding modes (MBM, green). Disorder-to-order binding mode is represented by the p53 oligomerisation domain (PDB:1c26, 325–356 residues, top left), which has a high probability of disorder-to-order transition (p_DO_) and low multiplicity of binding modes, thus preferentially samples ordered binding modes as observed (329–341 residues magenta). Context-dependent binding modes (top middle) are represented by the p53 transactivation region in complex with the general transcription factor TFIIH (41–62 residues, PDB:2ruk) and in complex HMGB1 (1–93 residues, PDB: 2ly4). The interacting regions (green: 49–55 residues, and blue: 56–60 residues) are predicted to have high MBM values, and a considerable probability to sample both ordered and disordered interactions. The disorder-to-disorder binding mode can be represented the p53 transactivator region (2–61 residues, PDB:5phd, top right) in complex with the TAZ2 domain of the CBP/p300 coactivator, through a short peptide motif (2–14 residues, teal) exhibiting high p_DD_ and low MBM values, in accord with the observed conformational heterogeneity. (**B**) The binding mode landscape characterises the interaction behavior. The binding mode landscape represents the binding mode (p_DD_, y axis) together with its variability, as characterised by MBM (x-axis). The lower left quadrant (*p*_DD_< 0.40; MBM < 0.55, red circles) represents the classical view of binding, with preferentially ordered interfaces, as represented by the p53 oligomerisation domain (PDB:1c26). The upper left quadrant (*p*_DD_≥ 0.60; MBM < 0.55, cyan pentagons) represents preferentially heterogeneous complexes, as represented by the p53 complex with TAZ2 of CBP/p300 (PDB:5phd). The lower right quadrant (*p*_DD_< 0.40; MBM ≥ 0.65, light blue triangles) represents polymorphic regions that form different structures in a context-dependent manner, for example amylogenic regions (45). The upper right quadrant (*p*_DD_≥ 0.60; MBM ≥ 0.65, green squares) represents disordered binding regions that can conditionally convert from disordered to ordered binding modes, like in case of aggregation hot-spots (21). Context-dependent binding modes are shown in the right part of the landscape, represented by 49–55 residues interacting with general transcription factor TFIIH (PDB:2ruk, green squares) and 56–60 residues interacting with HMGB1 (PDB: 2ly4, blue triangles). The more stable binding modes have low MBM values and can be found in the left part of the binding mode landscape. These include the disorder-to-order binding mode, represented by the 329–341 residues of the oligomerisation domain (PDB:1c26, magenta circles), and the disorder-to-disorder binding mode represented by the 2–14 residues in complex with with the TAZ2 domain of the CBP/p300 coactivator (PDB:5phd, cyan pentagons).

The binding mode landscape describes the interaction behavior of proteins by simultaneously characterising the binding mode and the MDM (18,20). The x-axis displays the MBM and the y-axis displays the probability to form disordered interactions (Figure 3B). These two parameters inform on the most likely binding mode and its sensitivity to different partners or cellular conditions. Protein regions that usually remain heterogeneous in their assemblies are located at the upper part of the landscape. Protein regions that prefer undergoing disorder-to-order transitions are located at the lower part of the landscape. Protein regions that exhibit similar binding modes in a variety of conditions are located in the left part of the landscape, and protein regions that change their binding modes with the cellular conditions can be found in the right part of the landscape (Figure 3B).

The regions that form disordered complexes with a variety of partners (upper left quadrant) often drive protein phase separation (9). In contrast, regions which tend form disordered assemblies, but can be induced to form ordered structures can be found at the upper right quadrant (21). These regions often serve as hot-spots of aggregation (22). Regions in the lower right quadrant may undergo disorder-to-order transitions upon binding, but can form polymorphic structures. These are typically the regions serving as amyloid cores (22). Finally, the regions in the lower left quadrant are regions that adopt a well-defined structure upon binding with all their interaction partners.

### Predicted interaction characteristics available from the FuzPred web server

**Figure 4.**
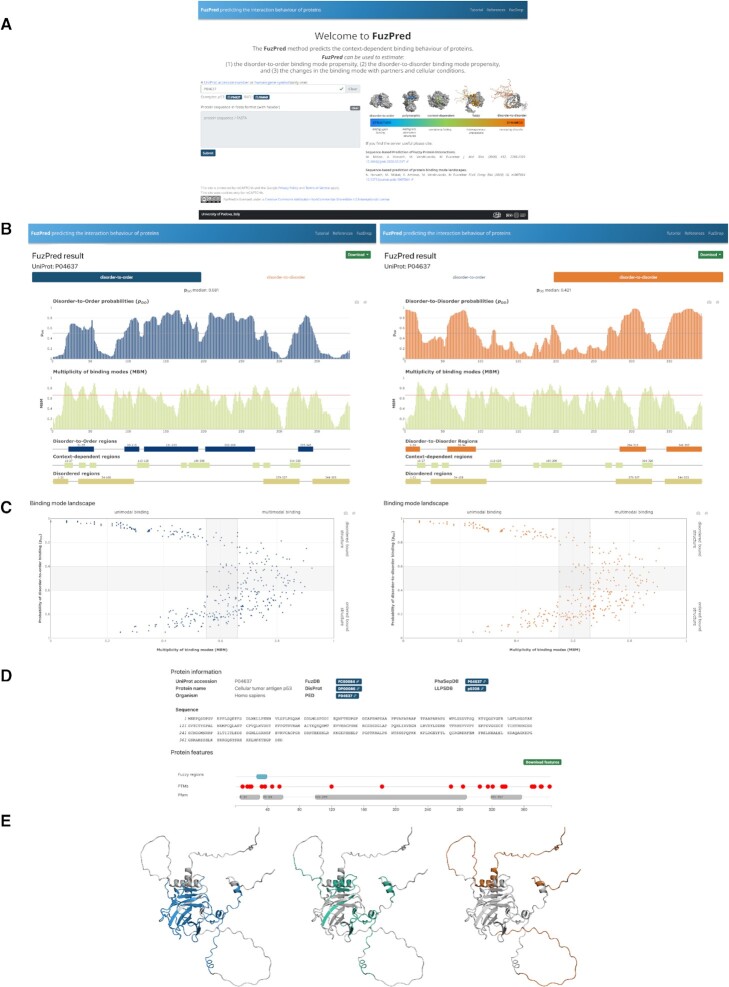
FuzPred server Input (**A**) and Result's (B–E) pages. The usage of the FuzPred server is illustrated using the sequence of the p53 tumor suppressor. (A) Input page. The input can be specified by the gene or UniProt identifier (P04637). Alternatively, the sequence can be provided, using only standard amino acids. In case of modified sequence, the cross-links with other databases, such as UniProt, Pfam will not be displayed on the Results page. (**B**) Prediction of binding mode and multiplicity of binding modes. The user may choose from the upper tabs to display the residue-specific probabilities of disorder-to-order (p_DO_, left) or disorder-to-disorder (p_DD_, right) transitions. In both cases, the multiplicity of binding modes (MBM) is displayed below. This graph, reflecting context-dependence of the interactions, does not depend on the binding mode analyzed. Regions undergoing disorder-to-order (left, blue) or disorder-to-disorder transitions (right, orange) are displayed under the MBM graph. In both cases, context-dependent regions (MBM ≥ 0.65, light green) and disordered regions by the Espritz algorithm (light brown) are also shown. (**C**) Binding mode landscape. The binding mode (y-axis: disorder-to-order, left; disorder-to-disorder, right) is displayed as a function of the likelihood of changing the binding mode (x-axis: multiplicity of binding modes, MBM). The characteristics of the four regions of the landscape are labelled, the grey areas have mixed (intermediate) properties. (**D**) Protein information. Cross-links to experimental databases of protein disorder and liquid-liquid phase separation, and sites of posttranslational modifications. (**E**) Visualisation of regions with different interaction characteristics. The disorder-to-order (left, blue), the disorder-to-disorder (orange, right) and the context-dependent (green, middle) are displayed on structured generated by AlphaFold.

All predictions are based on solely the protein sequence, which can be provided for the input as a UniProt code (23) or the FASTA file (Figure 4A). Importantly, no information on the partner identity is required. The results are displayed on a separate page.

### Prediction of the binding mode

The binding mode is characterised by the disorder-to-order or disorder-to-disorder transitions upon binding. The user can choose the type of structural transition using the two tabs on the top of the page (Figure 4B). Disorder-to-order transitions characterises the sequence based probability of ordering upon binding (*p*_DO_, blue) (Figure 4B). In case of disordered proteins, regions with *p*_DO_ ≥ 0.6 likely adopt a folded structure upon binding. In case of ordered proteins, regions with *p*_DO_ ≥ 0.6 rigidify upon binding. Disorder-to-disorder transitions characterises the sequence based probability of decreasing order upon binding (*p*_DD_, orange) (Figure 4B). Protein regions with *p*_DD_ ≥ 0.6 are likely remain or become heterogeneous conformational ensemble in the bound assembly. Folded proteins regions with *p*_DD_ ≥ 0.6 may unfold upon interactions. Protein regions with *p*_DO_ and *p*_DD_ in the range of 0.4–0.6 usually can sample both binding modes.

The graphs displaying the probabilities of disorder-to-order or disorder-to-disorder transitions are interactive (Figure 4B). Moving the cursor above the columns the graph show the *p*_DO_ or *p*_DD_ values belonging to each data point, and the identity the given residue. One can also zoom on selected regions of interest.

### Prediction of the multiplicity of binding modes (MBM)

The likelihood that a protein region samples multiple binding modes under different cellular conditions is shown in the second panel below the graphs on the binding mode predictions (Figure 4B). The MBM value is derived from the Shannon entropy computed from the binding mode distributions in the presence of a series of hypothetical partners (21), normalised into the range of [0,1] (14). Protein regions with MBM ≥0.65 are sensitive to the cellular context and are expected to sample multiple binding modes (21). Protein regions with MBM < 0.55 likely sample one binding mode. The MBM graph is interactive (Figure 4B) showing the identity of residues and the corresponding MBM values. The MBM graph is identical for the disorder-to-order or disorder-to-disorder transitions.

### Identification of protein regions with different interaction behaviors

Protein regions with particular interaction characteristics are defined below the binding mode and MBM graphs (Figure 4B). At least five consecutive amino acid residues with the same property are considered as a region, which is represented by a bar, with the same color as the corresponding graph. Disorder-to-order regions (DORs) are defined as *p_DO_* ≥ 0.6 (blue), disorder-to-disorder regions (DDRs) as *p*_DD_ ≥ 0.6 (orange). Context-dependent regions, defined as MBM ≥ 0.65. In addition, protein regions, predicted to be disordered (Espritz score ≥ 0.3085 (24)) in the unbound form are also shown. The boundaries of the regions are displayed above the bars as well as shown by moving the cursor above them.

### Binding mode landscape

The residue-specific interaction behaviors are shown on the binding mode landscape, which simultaneously displays the binding mode (y-axis) and the MBM, the multiplicity of binding modes (x-axis), which can be sampled in different cellular environments (Figure 4C). The MBM values are normalised into the range of [0,1]. All residues are represented by symbols and their identity is shown by moving the cursor above the symbols. The graph is divided into four sections, corresponding to different interaction behaviors (Figure 4C). The lower left quadrant corresponds to structured interaction elements that rigidify or adopt structure upon binding (low *p*_DD_, high *p*_DO_, low MBM). The upper left quadrant corresponds to protein regions that remain to be disordered under a wide variety of cellular conditions and partners (high *p*_DD_, low *p*_DO_, low MBM). These regions are distinguished in protein phase separation (9). The lower right quadrant displays the structured, polymorphic interaction elements (low *p*_DD_, high *p*_DO_, high MBM), for example amylogenic regions (22). The upper right quadrant shows those residues that dominantly sample disordered binding configurations, but can also be triggered to ordered bound states (high *p*_DD_, low *p*_DO_, high MBM). These residues are hot-spots for aggregation (22). The boundaries of the regions are marked by gray. Protein regions falling into the borderlines usually exhibit a mixture of behaviors.

### Sequence features and cross-links to other databases

The FuzPred server provides information on some sequence features that may be relevant to regulate binding characteristics. In this panel, the sequence corresponding to the UniProt code or the sequence provided is shown (25). Below the experimentally observed fuzzy regions, with validated functional impacts are displayed as derived from the FuzDB, the database of fuzzy interactions (4). Below post-translational modification sites (PTMs) derived from UniProt database are displayed by red dots (26). Positioning the cursor above the symbols will display the modified residue, the PTM type and the modifying enzyme. Evolutionary conserved protein domains, which are derived from the Pfam database (27,28), are shown in the last row (Figure 4D).

Crosslinks to experimental databases, such as on protein disorder (DisProt (29)), protein structural ensembles (PED (30)) and fuzzy interactions (FuzDB (4)) are provided below. As protein regions with disorder-to-disorder binding modes are distinguished in protein liquid-liquid phase separation (9), cross-links to PhaSepDB (31), LLPSDB (32) and PhaSePro (33) databases of liquid-liquid phase separation are displayed.

### Graphical representation of disorder-to-order, disorder-to-disorder and context-dependent regions

Protein regions with different interaction characteristics are visualised by Mol* (34) on the structures predicted by AlphaFold (35) (AF, Figure 4E), which are accessed by using 3D-Beacons network (36). Disorder-to-order regions are shown in blue and disorder-to-disorder regions in orange and context-dependent regions (MBM ≥ 0.65) in green (Figure 4E). In case the predicted structure is not available in the AlphaFold database (37), the user may carry out the structure predictions for the given sequence in a separate window (Figure 4E) through AlphaFold Colab provided by DeepMind and Google (38). The coordinates derived from the AlphaFold predictions (35), can then be uploaded to visualise the interaction properties estimated by FuzPred (Figure 4E). The AlphaFold structures show disordered regions as extended chains, which may significantly deviate from in particular in their bound states (39).

### Download options

The FuzPred prediction results, the residue-based *p*_DO_, *p*_DD_ and MBM values can be downloaded in .tsv format via the ‘Download’ tab on the top right of the page. The coordinates of the disorder-to-order, disorder-to-disorder regions and context-dependent regions can also be downloaded in .tsv format via the ‘Download’ tab on the top right of the page. The graph displaying the *p*_DO_, *p*_DD_ and MBM values together with the bar representation of the different interacting regions can be saved as an image by the camera icon below the ‘Download’ tab on the top of the page

A snapshot of the the colored AlphaFold structures representing protein regions of different interaction behaviors can be generated using the ‘Screenshot’ tab above the image, and the coordinates of the predicted structure can be downloaded. This option is useful for further graphical analysis including other features, for example posttranslational modification sites.

### Tutorial page and references

The Tutorial provides a detailed description of the results, which are shown in the results page. This page is organised similarly to the above sections of the article. On the right side a navigator bar facilitates orientation on the page. The References assembles the literature on fuzziness, organised in a thematic manner. A cross-link to the FuzDrop server (http://fuzdrop.bio.unipd.it) (40) is provided to further explore the liquid-liquid phase separation characteristics of the protein.

### FuzPred server application areas

The FuzPred server has four main application areas, describing different interaction behaviors (Table 1).

1. ***Identification of the binding modes of protein regions*.** The probabilities of the disorder-to-order (*p*_DO_) and disorder-to-disorder (*p*_DO_) transitions inform on the binding elements that adopt a well-defined structure upon binding and those that remain to be heterogeneous in the specific assembly.
2. ***Identification of protein regions with multiplicity of binding modes*.** The MBM values inform on the likelihood of sampling multiple binding modes under different cellular conditions. Context-sensitive residues can exhibit both ordered or disordered binding modes and can for example serve as posttranslational modification sites.
3. ***Identification of interaction behaviors: polymorphic regions and dynamic regulatory regions*.**Residues with high MBM and *p*_DO_ values can sample different ordered conformations and exhibit polymorphism in their complexes. Residues with high MBM and *p*_DD_ values are dominantly disordered, and can be induced to be ordered to form transient contact sites.
4. ***Identification of mutations inducing aggregation*.** Mutations that cause protein aggregation can induce either unfolding (deposition) or maturation of condensates (condensation). In the first case the mutation will shift the position in the binding landscape along the diagonal, whereas in the second case, the shift will be horizontal to the right, possibly in the lower part of the binding landscape.

**Table 1.** Application areas of the FuzPred web server

Problem	Parameter, threshold
Identification of recognition elements of disordered proteins	*p*_DO_≥ 0.60
Identification of protein regions forming fuzzy complexes	*p*_DD_≥ 0.60
Identification of context-dependent interaction sites (e.g. post-translational modification site)	MBM≥ 0.65
Identification of regions that can partition into protein condensates	*p*_DD_≥ 0.60
Identification of aggregation hot-spots within protein condensates	*p*_DD_≥ 0.60; MBM ≥ 0.65

## CONCLUSIONS

It is increasingly recognised that proteins exhibit complex interaction behaviors in cells by sampling a wide range of binding modes from ordered to disordered bound states, which may vary depending on the cellular conditions. The FuzPred web server provides a comprehensive description of the interaction behaviors by predicting both the binding mode and the multiplicity of binding modes without the need of specifying the binding partner. Thus, the FuzPred web server identifies regions that are dominantly structured or disordered in their complexes as well as context-sensitive sites that alternate between these binding modes. Such analysis, and the representation of the results on the structures predicted by AlphaFold, enables the identification of molecular recognition or regulatory sites as well as segments of proteins driving higher-order assemblies. The residue-based probabilities enable the analysis of mutants, and elucidating the impact of disease-associated mutations on protein interactions.

## DATA AVAILABILITY

The authors confirm that the data in the article are publicly available.
